# Ethnicity-Specific Association Between Ghrelin Leu72Met Polymorphism and Type 2 Diabetes Mellitus Susceptibility: An Updated Meta-Analysis

**DOI:** 10.3389/fgene.2018.00541

**Published:** 2018-11-14

**Authors:** Rong Huang, Sai Tian, Rongrong Cai, Jie Sun, Yanjue Shen, Shaohua Wang

**Affiliations:** ^1^Department of Endocrinology, Affiliated Zhongda Hospital of Southeast University, Nanjing, China; ^2^Medical School of Southeast University, Nanjing, China

**Keywords:** ghrelin, Leu72Met, type 2 diabetes mellitus, polymorphism, meta-analysis

## Abstract

**Background:** The Leu72Met polymorphism of ghrelin gene has been associated with genetic predisposition to type 2 diabetes mellitus (T2DM), while conclusions remain conflicting. Hence, we performed this updated meta-analysis to clarify the association between Leu72Met polymorphism and T2DM susceptibility.

**Methods:** Six electronic databases were consulted for articles published before 1 January, 2018. Pooled odds ratios (OR) and 95% confidence intervals (CI) were calculated under five genetic models to assess this association. We used *I*^2^-test and *Q* statistics to measure heterogeneity across the included studies. Subgroup analyses and publication bias were also performed.

**Results:** Thirteen case-control studies involving 4720 T2DM patients and 4206 controls were included in this meta-analysis. The overall results using fixed-effects models showed that Leu72Met polymorphism was significantly associated with an increased risk of T2DM under homozygous model (OR = 1.307, 95%CI 1.001–1.705, *p* = 0.049). Further subgroup analyses stratified by ethnicity revealed that the risk for T2DM was only increased in Asians (homozygous model: OR = 1.335, 95%CI 1.014–1.758, *p* = 0.040), while decreased in Caucasians (dominant model: OR = 0.788, 95%CI 0.635–0.978, *p* = 0.030; heterozygous model: OR = 0.779, 95%CI 0.626–0.969, *p* = 0.025; allelic model: OR = 0.811, 95%CI 0.661–0.995, *p* = 0.045). Funnel plots were basically symmetrical, and all *p*-values of Egger's test under five genetic models were >0.050, which indicated no evidence of publication bias.

**Conclusions:** Our results demonstrate that the Leu72Met polymorphism of ghrelin gene may be protective against T2DM in Caucasians, while predisposing to T2DM in Asians.

## Introduction

Type 2 diabetes mellitus (T2DM), the most common metabolic disease, is characterized by a progressive decline in pancreatic β-cell function and increase in insulin resistance. It is estimated that ~415 million adults across the world were diagnosed with diabetes in 2015, and this number is predicted to rise to 642 million by 2040 (Ogurtsova et al., 2017). Among these, T2DM accounts for 90 to 95%, which makes it a public health problem. However, detailed etiologies underlying T2DM remain unclear. Recently, researches on genetic polymorphisms become one of the most attention areas in the pathogenesis of T2DM, and some studies indicate that genetic polymorphisms have potential roles in the etiology of T2DM (Fuchsberger et al., 2016).

Ghrelin (GHRL), a unique 28-amino acid gastrointestinal peptide hormone, was first discovered by Kojima and Kangawa in 1999 as the endogenous ligand for the growth hormone secretagogue receptor (Kojima et al., 1999). There are two major forms of ghrelin in the blood, including acyl ghrelin and des-acyl ghrelin, in which about 80–90% of circulating ghrelin exists in des-acyl form, while only the acylated form was demonstrated to be biologically active (Hosoda et al., 2000). Both ghrelin and its receptor were found widely expressed in peripheral tissues and brain, thus exert important roles including stimulating gastric acid secretion, regulating glucose and lipid metabolism, and modulating learning and memory functions (Muller et al., 2015; Alamri et al., 2016; Hsu et al., 2016). Ghrelin also had influences on the stress response and reward processing, as well as, in regulating reproductive function (Sominsky et al., 2017). In addition, ghrelin played a principal role in inhibiting inflammation, increasing cardiac output, and chronic respiratory failure (Mosa et al., 2015; Matsumoto et al., 2017). Evidence from clinical studies suggested that serum ghrelin correlated negatively with body mass index (BMI), waist circumference, insulin resistance (IR), and metabolic syndrome (MS), which indicated that ghrelin may be involved in the occurrence of T2DM (Serra-Prat et al., 2009; Amini et al., 2012; Soriano-Guillen et al., 2016).

**Figure 1:**
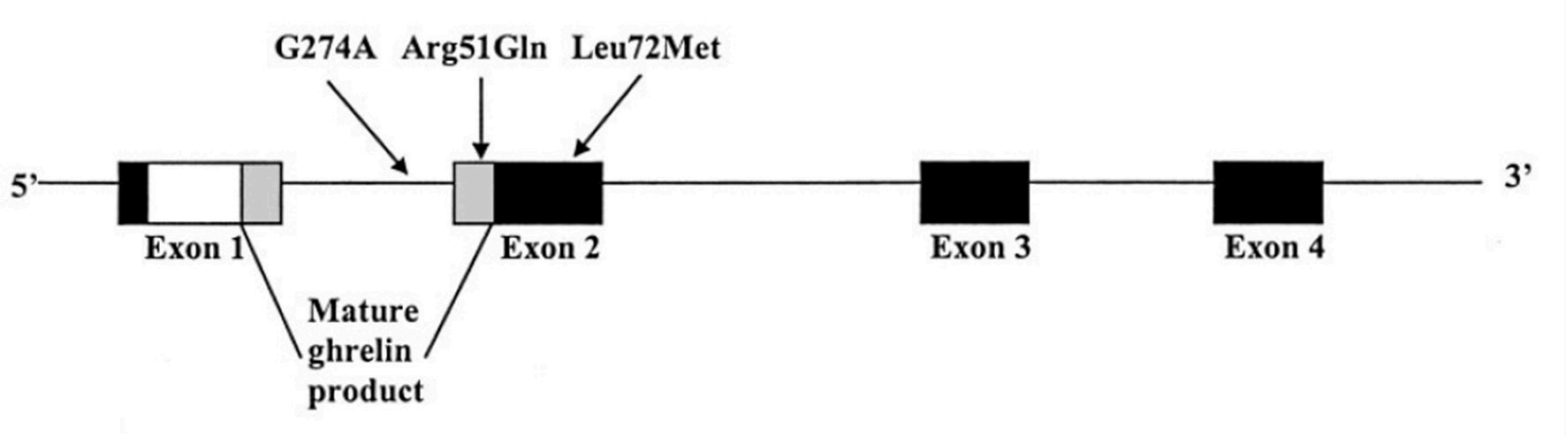
Structure of the preproghrelin gene and sites of the mutations detected.

The human ghrelin gene is located on chromosome 3p25–26 and comprises 4 exons and 3 introns (Ukkola et al., 2001). To date, several single nucleotide polymorphisms (SNPs) of the GHRL gene have been described, including Leu72Met (rs696217), Arg51Gln, and Gln90Leu (rs4684677) (Hinney et al., 2002). Among them, a common Leu72Met polymorphism was identified between the coding regions of mature ghrelin and obestatin encoded in exon 2 of the GHRL gene, and has been linked to several obesity-related phenotypes (Figure 1) (Ukkola et al., 2001). Previous studies showed that the Leu72Met polymorphism was related to obesity, insulin metabolism, and metabolic syndrome (Steinle et al., 2005; Kuzuya et al., 2006; Xu et al., 2008; Zavarella et al., 2008). As obesity and insulin metabolism are closely linked to T2DM, more and more studies have been performed in attempt to investigate the relationship between the Leu72Met polymorphism and T2DM risk across various counties (Larsen et al., 2005; Choi et al., 2006; Kim et al., 2006; Jiang et al., 2008; Xu and Xiang, 2008; Berthold et al., 2009; Garcia et al., 2009; Cui et al., 2010; Xiang et al., 2011; Zhang et al., 2011; Liu et al., 2012; Zhuang et al., 2014; Joatar et al., 2017). However, the results remain conflicting. Consequently, we conducted this meta-analysis to clarify the association between Leu72Met polymorphism and T2DM susceptibility.

## Materials and methods

This meta-analysis was conducted based on the methodology advocated by the Meta-analysis of Observational Studies in Epidemiology (MOOSE) guideline (Stroup et al., 2000).

### Literature search

Three English (PubMed, Cochrane Library, and Web of Science) and three Chinese (CNKI, Wanfang, and SinoMed) electronic databases were consulted for English and Chinese peer-reviewed articles published before 1 January 2018 regarding the association between ghrelin Leu72Met polymorphism and T2DM susceptibility. For the English databases, search strategy (“diabetes” or “T2DM”) and (“ghrelin” or “GHRL” or “Leu72Met” or “+408 C > A” or “rs696217”) and (“polymorphism” or “mutation” or “variant”) were applied. For the Chinese databases, search terms included “T2DM,” “ghrelin,” and “polymorphism.” Additional publications from the reference lists of original research articles and review articles were also searched. The papers were limited to humans and published in English or Chinese.

### Literature inclusion

Studies which meet the following inclusion criteria were considered eligible: (1) studies reporting the association between the Leu72Met polymorphism of ghrelin gene and T2DM risk; (2) case–control study design; and (3) providing sufficient genotype data that could calculate odds ratios (ORs) and 95% confidence intervals (CIs). The exclusion criteria were: (1) family or sibling pairs based studies; (2) editorials, case reports, reviews or meta-analyses, and (3) studies without detailed genotyping data. In addition, if there were duplicate publications from the same population, only the study with larger sample size and comprehensive data was included in our meta-analysis.

### Data extraction

For the included studies, data were independently extracted by two authors (Huang R and Tian S) as follows: the first author's name, year of publication, country, ethnicity, sample size of cases and controls, diagnostic criteria for T2DM, genotyping method, genotype and allele distributions of cases and controls, and controls with Hardy–Weinberg equilibrium (HWE) or not. The controversy about one article was discussed and resolved by a third reviewer (Cai RR).

### Quality assessment

The Newcastle-Ottawa quality assessment scale (NOS) was used to evaluate the methodological quality of the included case-control studies, which consists of the following three aspects: (1) selection of study subjects: 0–4 star, (2) comparability of study subjects: 0–2 star, and (3) exposure or outcomes: 0–3 star (Cook and Reed, 2015). The total score ranges from zero star to nine stars, and studies achieving 6 stars or more were considered as high quality.

### Statistical analysis

The pooled OR with a 95% CI was used to estimate the strength of the association between ghrelin Leu72Met polymorphism and T2DM risk under different comparison models, including dominant model (Leu72Met + Met72Met vs. Leu72Leu), recessive model (Met72Met vs. Leu72Met + Leu72Leu), homozygous model (Met72Met vs. Leu72Leu), heterozygous model (Leu72Met vs. Leu72Leu), and allelic model (Met72 + vs. Leu72+). Subsequently, we performed subgroup analyses to evaluate the effect of Leu72Met polymorphism on T2DM susceptibility under above-mentioned five genetic models according to the ethnicity of included populations. Heterogeneity across the included studies was assessed via *Q* statistics and *I*^2^-test. If the data showed no heterogeneity (*I*^2^ < 50% or *P*_Q_ ≥ 0.1), fixed-effects models were used; otherwise, random-effects models were applied. Sensitivity analysis was carried out by removing each study in sequence to assess the stability of the results. Additionally, Begg's funnel plots and Egger's regression test were used for investigating the potential publication bias. The results were considered statistically significant if the 2-tailed *p*-value was below 0.050. All the above-mentioned statistical analyses were performed with STATA software (Version 11.0) (College Station, TX, USA).

## Results

### Characteristics of included studies

**Figure 2:**
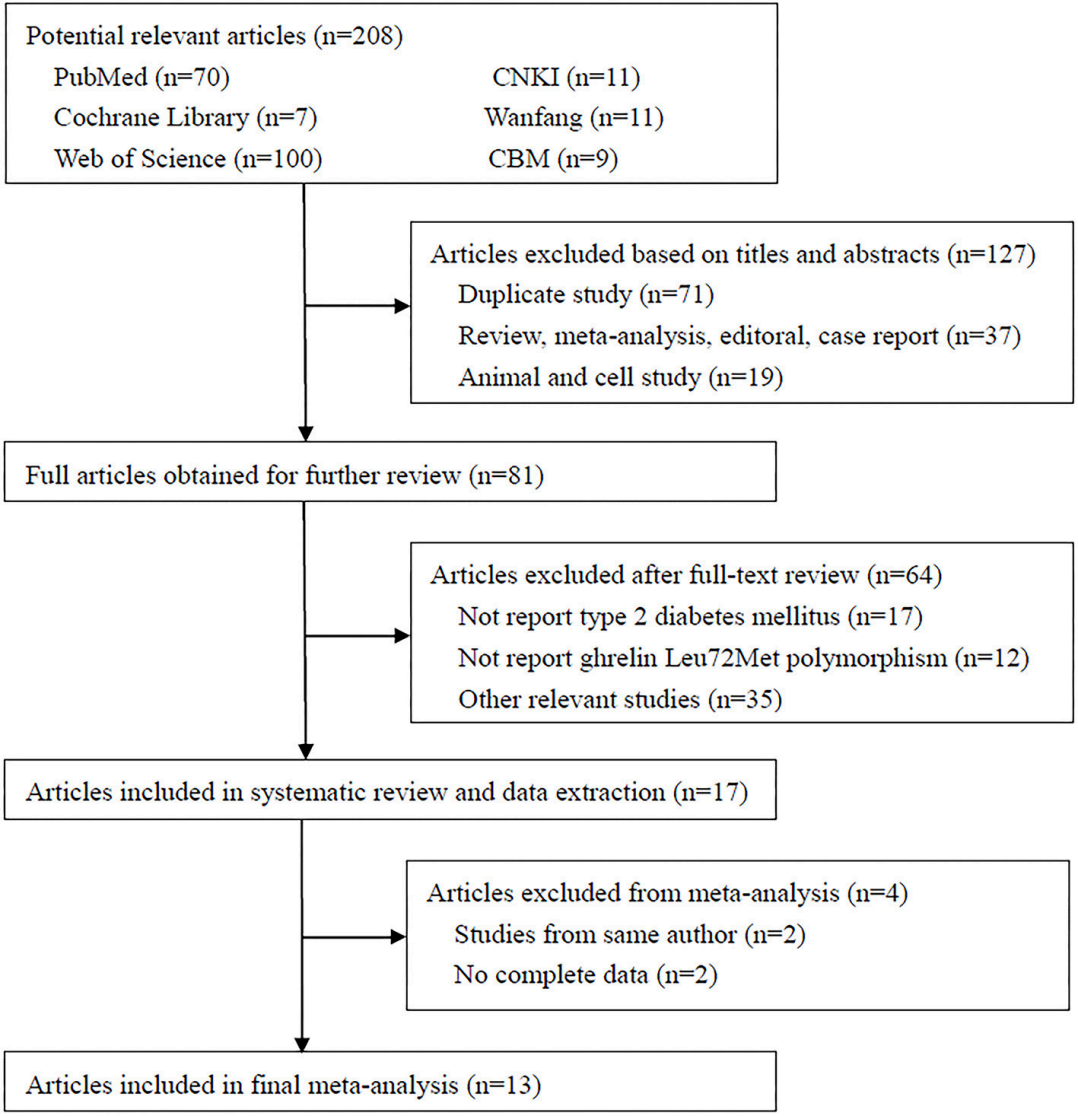
Flow chart of literature search.

A total of 208 studies were identified in the literature search using the above-mentioned search strategies (Figure 2). After the removal of duplicated literatures and articles that did not meet the inclusion criteria, 13 studies involving 4720 T2DM patients and 4,206 controls were finally included in this meta-analysis (Larsen et al., 2005; Choi et al., 2006; Kim et al., 2006; Jiang et al., 2008; Xu and Xiang, 2008; Berthold et al., 2009; Garcia et al., 2009; Cui et al., 2010; Xiang et al., 2011; Zhang et al., 2011; Liu et al., 2012; Zhuang et al., 2014; Joatar et al., 2017). Of these selected studies, eight studies were published in English (Larsen et al., 2005; Choi et al., 2006; Kim et al., 2006; Berthold et al., 2009; Garcia et al., 2009; Liu et al., 2012; Zhuang et al., 2014; Joatar et al., 2017), while the other five were published in Chinese (Jiang et al., 2008; Xu and Xiang, 2008; Cui et al., 2010; Xiang et al., 2011; Zhang et al., 2011). Based on the ethnicity of included studies, three studies were performed in Caucasians (Larsen et al., 2005; Berthold et al., 2009; Garcia et al., 2009), nine studies were conducted in Asians (Choi et al., 2006; Kim et al., 2006; Jiang et al., 2008; Xu and Xiang, 2008; Cui et al., 2010; Xiang et al., 2011; Zhang et al., 2011; Liu et al., 2012; Zhuang et al., 2014), and one study was in Arabians (Joatar et al., 2017). Six studies diagnosed T2DM with the World Health Organization (WHO) criteria (Larsen et al., 2005; Choi et al., 2006; Kim et al., 2006; Xiang et al., 2011; Zhang et al., 2011; Liu et al., 2012; Joatar et al., 2017), three studies employed the American Diabetes Association (ADA) criteria (Jiang et al., 2008; Garcia et al., 2009; Zhuang et al., 2014), two studies were defined as oral glucose tolerance test (OGTT) or any usage of antidiabetic medications (Xu and Xiang, 2008; Berthold et al., 2009), and one study didn't provide definite criteria (Cui et al., 2010). All included studies used blood samples for genotyping, with the majority of them using the polymerase chain reaction-restriction fragment length polymorphism (PCR-RFLP) method (Kim et al., 2006; Jiang et al., 2008; Xu and Xiang, 2008; Berthold et al., 2009; Cui et al., 2010; Xiang et al., 2011; Zhang et al., 2011), others using TaqMan (Choi et al., 2006; Zhuang et al., 2014; Joatar et al., 2017), PCR-single strand conformation polymorphism (PCR-SSCP) (Larsen et al., 2005), PCR-denaturing high-performance liquid chromatography (PCR-DHPLC) (Liu et al., 2012) or matrix-assisted laser desorption/ionization time of flight mass spectrometry (MALDI-TOF-MS) method (Garcia et al., 2009). The control samples from 12 included studies followed the HWE (Larsen et al., 2005; Choi et al., 2006; Kim et al., 2006; Jiang et al., 2008; Xu and Xiang, 2008; Berthold et al., 2009; Garcia et al., 2009; Cui et al., 2010; Zhang et al., 2011; Liu et al., 2012; Zhuang et al., 2014; Joatar et al., 2017), while only one did not follow it (Xiang et al., 2011). According to the NOS criteria, 12 studies were considered as high quality (Larsen et al., 2005; Choi et al., 2006; Kim et al., 2006; Jiang et al., 2008; Xu and Xiang, 2008; Berthold et al., 2009; Garcia et al., 2009; Xiang et al., 2011; Zhang et al., 2011; Liu et al., 2012; Zhuang et al., 2014; Joatar et al., 2017), and one study was low quality (Cui et al., 2010). Table 1 summarized the characteristics of each included study in details.

**Table 1:** Study characteristics from included studies in the meta-analysis.

**References**	**Country**	**Ethnicity**	**Sample size (case/control)**	**T2DM diagnostic criteria**	**Genotyping method**	**Case**			**Control**			**Controls with HWE**	**Score**
						**LL**	**LM**	**MM**	**LL**	**LM**	**MM**		
Larsen et al., 2005	Denmark	Caucasian	528/229	WHO (1999); (Alberti and Zimmet, 1998)	PCR-SSCP	455	71	2	194	33	2	Yes	6
Choi et al., 2006	Korea	Asian	758/636	WHO (1999); (Alberti and Zimmet, 1998)	TaqMan	518	215	25	429	185	22	Yes	7
Kim et al., 2006	Korea	Asian	206/80	WHO (1999); (Alberti and Zimmet, 1998)	PCR-RFLP	133	65	8	54	23	3	Yes	7
Jiang et al., 2008	China	Asian	252/83	ADA (2003); (The Expert Committee on the Diagnosis and Classification of Diabetes Mellitus, 2003)	PCR-RFLP	151	96	5	55	28	0	Yes	6
Xu and Xiang, 2008	China	Asian	202/333	OGTT	PCR-RFLP	143	57	2	251	80	2	Yes	9
Berthold et al., 2009	Germany	Caucasian	420/430	OGTT or if receiving antidiabetic therapy	PCR-RFLP	377	40	3	364	65	1	Yes	7
Garcia et al., 2009	France	Caucasian	600/743	ADA (1997); (The Expert Committee on the Diagnosis and Classification of Diabetes Mellitus, 1997)	MALDI-TOF-MS	523	75	2	633	108	2	Yes	6
Cui et al., 2010	China	Asian	102/95	NA	PCR-RFLP	71	25	6	68	27	0	Yes	5
Xiang et al., 2011	China	Asian	316/203	WHO (1999); (Alberti and Zimmet, 1998)	PCR-RFLP	272	37	7	179	21	3	No	8
Zhang et al., 2011	China	Asian	138/113	WHO (1999); (Alberti and Zimmet, 1998)	PCR-RFLP	82	51	5	80	30	3	Yes	8
Liu et al., 2012	China	Asian	864/877	WHO (1999); (Alberti and Zimmet, 1998)	PCR-DHPLC	463	336	65	472	353	52	Yes	6
Zhuang et al., 2014	China	Asian	238/291	ADA (2010); (American Diabetes Association, 2010)	TaqMan	146	70	12	197	85	9	Yes	8
Joatar et al., 2017	Saudi Arabia	Arab	96/93	WHO (1998); (Alberti and Zimmet, 1998)	TaqMan	89	7	0	84	8	1	Yes	6

*T2DM, type 2 diabetes mellitus; LL, Leu72Leu; LM, Leu72Met; MM, Met72Met; HWE, Hardy-Weinberg equilibrium; WHO, World Health Organization; ADA, American Diabetes Association; OGTT, oral glucose tolerance test; NA, not available; PCR-SSCP, Polymerase Chain Reaction-Single Strand Conformation Polymorphism; PCR-RFLP, Polymerase Chain Reaction-Restriction Fragment Length Polymorphism; MALDI-TOF-MS, Matrix-Assisted Laser Desorption/Ionization Time of Flight Mass Spectrometry; PCR-DHPLC, Polymerase Chain Reaction-Denaturing High-Performance Liquid Chromatography*.

### Synthesis analyses

**Figure 3:**
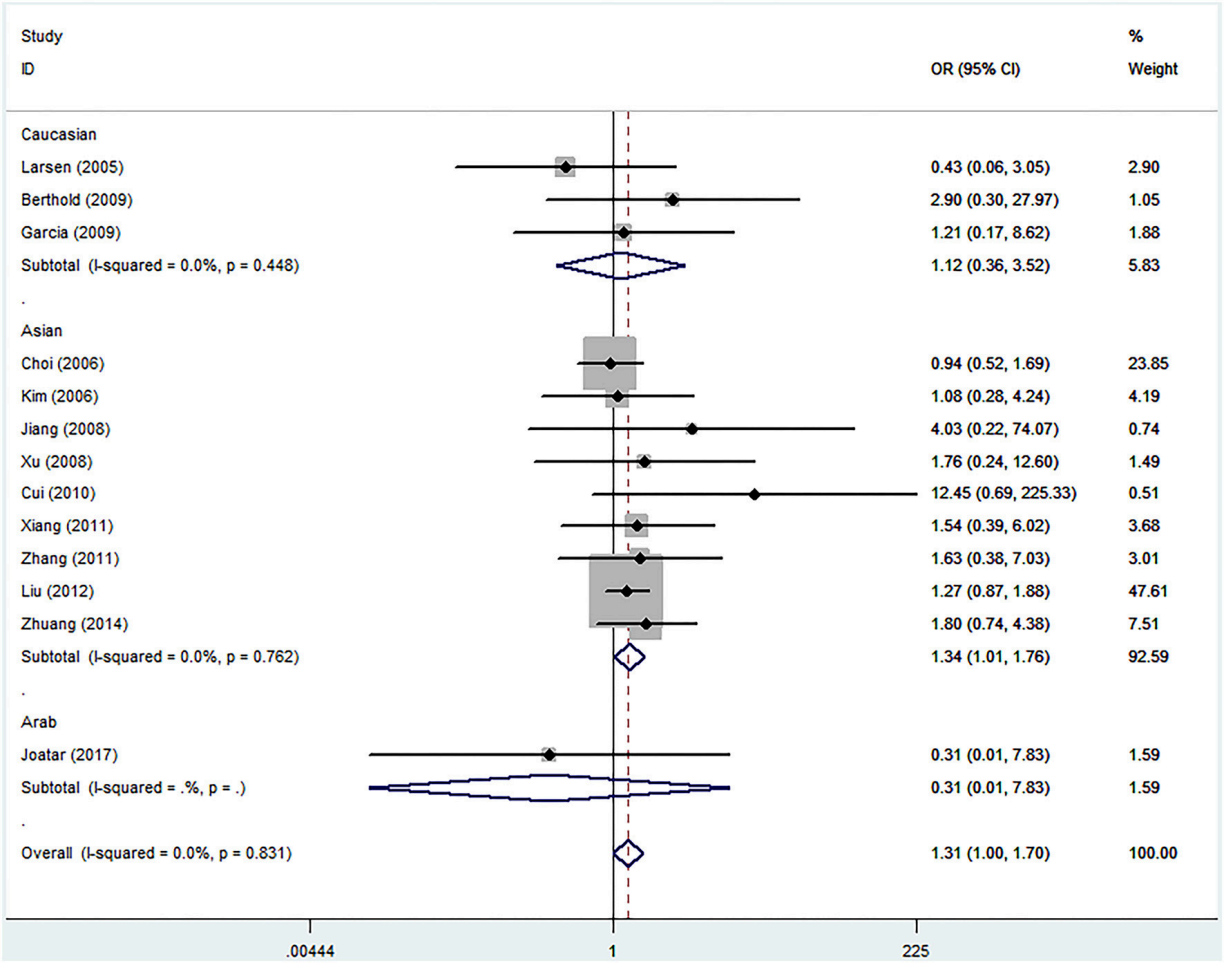
Forest plots of ghrelin Leu72Met polymorphism and risk of type 2 diabetes mellitus in homozygous model (fixed-effects model) (OR = 1.307, 95%CI = 1.001–1.705, *p* = 0.049).

Meta-analyses under five genetic models are shown in Table 2. No evidences of heterogeneity were found in our study, thus fixed-effects models were used and the pooled results showed that the Leu72Met polymorphism of ghrelin gene was significantly associated with an increased risk of T2DM under homozygous model (OR = 1.307, 95%CI = 1.001–1.705, *p* = 0.049) (Figure 3). Further subgroup analyses stratified by ethnicity revealed that T2DM risk was only increased in Asians (homozygous model: OR = 1.335, 95%CI 1.014–1.758, *p* = 0.040), while decreased in Caucasians (dominant model: OR = 0.788, 95%CI 0.635–0.978, *p* = 0.030; heterozygous model: OR = 0.779, 95%CI 0.626–0.969, *p* = 0.025; and allelic model: OR = 0.811, 95%CI 0.661–0.995, *p* = 0.045) (Table 2).

**Table 2:** Meta-analysis and heterogeneity test of the Ghrelin Leu72Met polymorphism and T2DM susceptibility.

**Genetic model**	**Subgroup**	**OR (95% CI)**	***p***	***I*^2^ (%)**	***P_*Q*_***
Dominant	Overall	1.010 (0.914–1.115)	0.847	15.2	0.291
	Ethnicity				
	Caucasian	**0.788 (0.635–0.978)**	**0.030**	0.0	0.436
	Asian	1.085 (0.969–1.214)	0.156	0.0	0.690
	Arab	0.734 (0.262–2.060)	0.557	–	–
Recessive	Overall	1.124 (0.867–1.457)	0.377	0.0	0.663
	Ethnicity				
	Caucasian	1.161 (0.371–3.630)	0.797	0.0	0.429
	Asian	1.134 (0.868–1.483)	0.357	0.0	0.521
	Arab	0.320 (0.013–7.943)	0.486	–	–
Homozygous	Overall	**1.307 (1.001–1.705)**	**0.049**	0.0	0.831
	Ethnicity				
	Caucasian	1.125 (0.359–3.524)	0.840	0.0	0.448
	Asian	**1.335 (1.014–1.758)**	**0.040**	0.0	0.762
	Arab	0.315 (0.013–7.833)	0.481	–	–
Heterozygous	Overall	0.983 (0.888–1.090)	0.749	11.6	0.329
	Ethnicity				
	Caucasian	**0.779 (0.626–0.969)**	**0.025**	14.5	0.310
	Asian	1.054 (0.937–1.184)	0.382	0.0	0.695
	Arab	0.826 (0.287–2.377)	0.723	–	–
Allelic	Overall	1.037 (0.952–1.130)	0.403	17.6	0.267
	Ethnicity				
	Caucasian	**0.811 (0.661–0.995)**	**0.045**	0.0	0.604
	Asian	1.098 (0.999–1.208)	0.053	0.0	0.657
	Arab	0.666 (0.248–1.7888)	0.420	–	–

*T2DM, type 2 diabetes mellitus; OR, odd ratio; CI, confidence interval. Bold values, significant differences*.

### Sensitivity analyses

Sensitivity analyses were performed by sequentially removing one study each time to explore the effect of individual data on the pooled effects. The significance of ORs was not altered excessively through omitting any single study in all compared genetic models, except for the homozygous model, in which, following the exclusion of the Jiang et al. (2008), Xu and Xiang (2008), Berthold et al. (2009), Garcia et al. (2009), Cui et al. (2010), Xiang et al. (2011), Zhang et al. (2011), Liu et al. (2012), Zhuang et al. (2014) studies, the overall OR became insignificant (OR = 1.286, 95%CI 0.984–1.681, *p* = 0.065; OR = 1.300, 95%CI 0.994–1.700, *p* = 0.056; OR = 1.290, 95%CI 0.986–1.686, *p* = 0.063; OR = 1.308, 95%CI 1.000–1.711, *p* = 0.050; OR = 1.250, 95%CI 0.955–1.636, *p* = 0.104; OR = 1.298, 95%CI 0.989–1.702, *p* = 0.060; OR = 1.297, 95%CI 0.989–1.700, *p* = 0.060; OR = 1.336, 95%CI 0.926–1.928, *p* = 0.122; OR = 1.267, 95%CI 0.959–1.674, *p* = 0.096, respectively) (Table 3).

**Table 3:** Sensitivity analysis by excluding individual study in each genetic model.

**References**	**Dominant model**		**Recessive model**		**Homozygous model**		**Heterozygous model**		**Allelic model**	
	**OR (95% CI)**	***p***	**OR (95% CI)**	***p***	**OR (95% CI)**	***p***	**OR (95% CI)**	***p***	**OR (95% CI)**	***p***
Larsen et al., 2005	1.017 (0.918–1.126)	0.749	1.143 (0.879–1.485)	0.319	**1.333 (1.019–1.744)**	**0.036**	0.987 (0.888–1.097)	0.810	1.045 (0.958–1.141)	0.320
Choi et al., 2006	1.022 (0.915–1.142)	0.698	1.088 (0.814–1.454)	0.570	**1.421 (1.054–1.917)**	**0.021**	0.988 (0.882–1.108)	0.841	1.055 (0.959–1.161)	0.268
Kim et al., 2006	1.006 (0.909–1.112)	0.913	1.128 (0.866–1.469)	0.373	**1.316 (1.004–1.726)**	**0.047**	0.978 (0.881–1.086)	0.680	1.035 (0.949–1.129)	0.440
Jiang et al., 2008	1.000 (0.903–1.106)	0.994	1.146 (0.882–1.489)	0.307	1.286 (0.984–1.681)	0.065	0.974 (0.877–1.081)	0.619	1.028 (0.942–1.122)	0.535
Xu and Xiang, 2008	0.995 (0.898–1.102)	0.919	1.136 (0.874–1.477)	0.339	1.300 (0.994–1.700)	0.056	0.967 (0.869–1.075)	0.533	1.026 (0.940–1.121)	0.562
Berthold et al., 2009	1.041 (0.940–1.153)	0.443	1.106 (0.851–1.437)	0.451	1.290 (0.986–1.686)	0.063	1.017 (0.914–1.130)	0.761	1.060 (0.971–1.158)	0.194
Garcia et al., 2009	1.030 (0.928–1.144)	0.580	1.122 (0.864–1.458)	0.388	1.308 (1.000–1.711)	0.050	1.002 (0.899–1.117)	0.973	1.055 (0.964–1.153)	0.245
Cui et al., 2010	1.008 (0.911–1.114)	0.884	1.189 (0.913–1.548)	0.200	1.250 (0.955–1.636)	0.104	0.986 (0.889–1.094)	0.791	1.030 (0.945–1.124)	0.499
Xiang et al., 2011	1.003 (0.907–1.110)	0.950	1.149 (0.881–1.497)	0.306	1.298 (0.989–1.702)	0.060	0.978 (0.881–1.085)	0.674	1.031 (0.945–1.125)	0.488
Zhang et al., 2011	0.991 (0.896–1.097)	0.867	1.141 (0.876–1.486)	0.328	1.297 (0.989–1.700)	0.060	0.964 (0.869–1.070)	0.495	1.023 (0.937–1.116)	0.610
Liu et al., 2012	1.010 (0.899–1.135)	0.867	0.991 (0.692–1.420)	0.961	1.336 (0.926–1.928)	0.122	0.988 (0.876–1.114)	0.847	1.032 (0.930–1.146)	0.552
Zhuang et al., 2014	0.998 (0.900–1.106)	0.964	1.078 (0.821–1.415)	0.589	1.267 (0.959–1.674)	0.096	0.974 (0.876–1.083)	0.629	1.024 (0.937–1.120)	0.596
Joatar et al., 2017	1.013 (0.917–1.119)	0.801	1.136 (0.875–1.474)	0.339	**1.323 (1.012–1.728)**	**0.040**	0.985 (0.889–1.092)	0.774	1.041 (0.955–1.134)	0.362

*OR, odd ratio; CI, confidence interval. Bold values, significant differences*.

### Publication biases

Funnel plot, Begg's test and Egger's test were performed to evaluate whether there exist possible publication biases. The funnel plots appeared to be basically symmetrical, and all *p*-values of Begg's test and Egger's test were >0.050, which indicated no evidences of publication biases in the current meta-analysis (data not shown).

## Discussion

T2DM is a multifactorial disorder attributing to the coexistence of a variety of genetic and environmental factors (Abdullah et al., 2017). In the past decade, over 70 loci in the human genome have been identified as potential risk factors in T2DM by genome-wide association studies (GWAS), including transcription factor-7-like 2 (TCF7L2), forkhead box A1 (FOXA1), and A2 (FOXA2), hepatocyte nuclear factor 4alpha (HNF4A) and high-mobility group AT-hook 1 (HMGA1) (Pullinger et al., 2014; Cheng et al., 2017). The association between Leu72Met polymorphism and T2DM susceptibility has been studied for several years, however, the results are still controversial. As previous single study conducted in a small sample size may limit the persuasion, here we carried out this meta-analysis to clarify the association between the Leu72Met polymorphism and T2DM susceptibility. The results revealed that the Leu72Met polymorphism was significantly associated with an increased risk of T2DM under homozygous model. Further subgroup analyses stratified by ethnicity indicated that T2DM risk was only increased in Asians, while decreased in Caucasians and unchanged in Arabians.

The Leu72Met mutation lies outside the region in which the mature ghrelin product is encoded and does not change the sequence, which makes its functional significance still unknown (Zavarella et al., 2008). Study from Kim et al. suggested that the variant may alter messenger RNA stability or protein processing, therefore, ghrelin secretion or activity could be modified rather than change the circulating level of ghrelin (Kim et al., 2006). In 2002, Ukkola et al. showed that subjects with Met72Met genotype had lower BMI than those with Leu72Leu genotype (Ukkola et al., 2002). Studies from 1420 Caucasian subjects also demonstrated that Met72 variant of the Leu72Met polymorphism exerted protective role in IR, which was evaluated by homeostasis model assessment of insulin resistance (HOMA-IR) index (Zavarella et al., 2008). Moreover, there was a tendency toward higher ghrelin levels in the Met72+ variant rather than in the Leu72Leu in T2DM subjects (Berthold et al., 2009), and ghrelin concentrations were found to correlate negatively with the prevalence of T2DM (Poykko et al., 2003). All the above findings support the result of our meta-analysis, which displayed a protective effect of the ghrelin Leu72Met polymorphism against T2DM risk in Caucasians.

An important finding was shown in this meta-analysis that ethnicity-specific association of the ghrelin Leu72Met polymorphism with T2DM risk between Asians, Caucasians and Arabians. Although the exact mechanism was not clear, several possible explanations may account for this ethnic difference. First, subjects from Asia, Caucasus, and Arab have different genetic background, which could at least partly explain the different observations. Previous studies found that the frequencies of Met72 allele were 21.88% in Japanese (*n* = 64) (Yamawaki et al., 2015), 18.13% in Koreans (*n* = 80) (Kim et al., 2006), 16.92% in Chinese (*n* = 1,962) (You et al., 2017), 13.85% in Finnish (*n* = 509) (Laurila et al., 2014), 7.8% in Danish (*n* = 2,134) (Bing et al., 2005) and 4.3% in Italians (*n* = 119) (Monteleone et al., 2007). In the current study, the distribution of the Met72 allele varied substantially between the three ethnic groups, with a prevalence of 18.81% in Asians, 7.70% in Caucasians, and 4.50% in Arabians. Second, T2DM is a complex disorder which was attributed to the interactions of multiple genetic and environmental factors. It is possible that different environmental exposures, life styles, and socioeconomic statuses may modify the individual's susceptibility in a different way (Langenberg et al., 2014; Raghavan et al., 2015). Furthermore, the result that protective effect of Leu72Met polymorphism on Caucasian T2DM subjects was draw from the synthesis analysis of three studies; the harmful effect on T2DM susceptibility among Asians was concluded according to nine studies, among which five were written in Chinese; and the neutral effect on Arabian T2DM subjects was only based on one study. The NOS scores of the included studies ranged from 5 to 9, which suggest the qualities were not so high. Therefore, the small sample size, the relatively low quality and the Chinese studies could all decrease the statistical power and limit the interpretation of our findings to some extent.

The association between ghrelin Leu72Met polymorphism and T2DM susceptibility has been assessed by a previous meta-analysis, which included six case-control studies (Liao et al., 2013). The results concluded that there was a decreased T2DM risk in subjects with Met72+ genotypes in Caucasians; while no association of the ghrelin Leu72Met polymorphism with T2DM risk was found in Asians. In the current meta-analysis of data from 13 case-control studies, we found that T2DM risk was increased in subjects with Met72Met genotype, and further subgroup analysis indicated the increased T2DM risk was only in Asians. Moreover, the decreased risk for T2DM in Caucasians were also showed in heterozygous and allelic models. The differences may be attributed to the inadequate number of included studies in Liao et al. study (Liao et al., 2013). Moreover, sensitivity analyses were performed in our study, which suggested a relatively stable result and made our results more reliable than previous meta-analysis. The results of this study may provide a specifically protective or hazardous effect of the ghrelin Leu72Met polymorphism on T2DM susceptibility among Caucasians, Asians, and Arabians.

Nevertheless, there were also several limitations that should be addressed in the present study. First, the number of studies and the sample size for Caucasian origin were relatively small, which could cause type II error and might have insufficient power for the detection of pooled effects. For example, if we set the significance level (α) as 0.05, the total sample size of 2,950 subjects with 1,548 cases and 1,402 controls and 6.46 and 7.70% of Met72 allele in cases and controls only provides 25.90% power to detect the OR of 0.79 in the dominant model in Caucasians. Moreover, sensitivity analysis of this meta-analysis also indicated that the overall results were somewhat unstable. Second, we only included the studies performed in Caucasians, Asians, and Arabians, which limited the application to a certain degree. Third, the overall results of our study were based on individual unadjusted OR because we had no access to the original data, such as BMI and IR. Additionally, gene-gene interaction and T2DM-related phenotypes were not taken into account because of limited date addressing these issues.

In conclusion, our results demonstrated that the Leu72Met polymorphism of ghrelin gene is protective against T2DM in Caucasians, while predisposing to T2DM in Asians. Since the low power of detecting effects and other potential biases and confounders can't be ruled out completely in this meta-analysis, further studies based on a large sample size are needed to verify the current findings.

## Author contributions

SW and RH designed the study. RH, ST, and RC collected the data. RH, JS, and YS performed the analyses. RH wrote the first draft. SW and ST checked the manuscript and revised it. All authors approved the final submission.

### Conflict of interest statement

The authors declare that the research was conducted in the absence of any commercial or financial relationships that could be construed as a potential conflict of interest.
